# Engineering Protein Stability with Small Molecules: A Review of the ecDHFR Destabilizing Domain System

**DOI:** 10.1021/acschembio.6c00466

**Published:** 2026-06-09

**Authors:** Miriam Ruhinda, Roenick P. Olmo, Bianca C. Burini

**Affiliations:** † Florida Medical Entomology Laboratory, University of Florida - IFAS, Vero Beach, Florida 32962, United States; ‡ 27083 CNRS UPR9022, Inserm U1257, Université de Strasbourg, Strasbourg 67084, France

## Abstract

The *E. coli* dihydrofolate reductase (ecDHFR) destabilizing domain (DD) is a versatile post-translational tool for the conditional control of protein stability via ligand-induced stabilization. In this system, a DD-tagged protein is rapidly degraded by the proteasome unless stabilized by the antibiotic trimethoprim (TMP), allowing for conditional control of protein abundance. The ecDHFR-DD system has been successfully applied across diverse biological systems, including yeast, invertebrate models such as *Drosophila*, and mammalian cells, to study a broad spectrum of cellular and developmental processes. Compared with DNA- and RNA-based regulatory approaches, post-translational systems offer faster response times and more precise control, making them valuable for processes that require tight, reversible regulation. In this review, we synthesize current knowledge on the mechanisms, performance, and optimization of the ecDHFR-DD system across organisms and evaluate its advantages and limitations relative to most conditional gene expression systems. We also highlight emerging opportunities for applying the system across diverse areas, ranging from functional genomics and synthetic biology to biomedical research. Additionally, we discuss its potential application in applied biological systems, such as pest and vector management, positioning the ecDHFR-DD system as a broadly applicable platform for the precise and tunable control of protein function across diverse disciplines.

## Graphical abstract

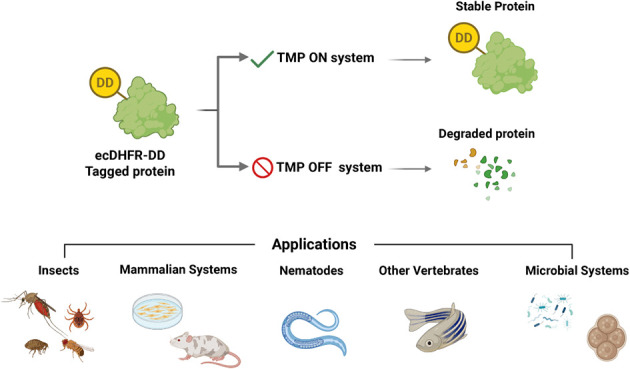

## 1 Introduction

Precise regulation of gene and protein functions is a fundamental challenge in modern biology, driving progress in functional genomics, synthetic biology, and biomedical research. Conditional gene expression systems play a crucial role in addressing this challenge by enabling the spatial and temporal regulation of genes of interest in response to defined stimuli,^1,2^ allowing researchers to better understand gene function during development.^1,3^ These systems enable genes to be turned on or off in response to various factors, such as chemicals, specific promoters, temperature, or light, in a reversible and dose-dependent manner.^4^ Beyond basic research, such regulatory tools are important in the regulation of cellular signaling pathways,^5^ the control of protein therapeutics in gene therapy,^6^ and understanding the possible roles of genes and associated proteins, as well as phenotypes, through protein modulation.^7^

A wide range of strategies has been developed to achieve conditional regulation at multiple levels of gene expression, including DNA, RNA, and protein.^8,9^ At the DNA level, genome editing and engineering can be achieved through various approaches, such as the CRISPR/Cas9 system which can induce mutations ranging from single nucleotides to large insertions in the genome. Additionally, site-specific recombination systems, such as Cre/loxP, FLP/FRT, and φC31 integrase, enable the conditional activation, inactivation, or rearrangement of genes by excising, inverting, or integrating DNA sequences in response to an inducer,^10^ and transposable elements that can be used for the random insertion of several base pairs into a genome and are also involved in the control of gene expression at the transcriptional and/or post-transcriptional levels.^11^ These changes at the DNA level are often irreversible but can be designed for temporal or tissue-specific control.^12^ Transcription processes can also be manipulated. Binary systems such as GAL4/UAS, Tet-On/Tet-Off, the Q-system, and hormone-responsive systems control transcriptional expression in response to the addition or removal of small molecules or hormones^7,13,14^ (see Table 1 for DNA-level regulation systems). Most recombination technology is used in lineage tracing as well as gene function studies in biology.^15,16^ While DNA and transcription-level approaches are highly versatile and widely adopted, they often exhibit slower response times and, in some cases, irreversible effects. Most transcriptional binary systems, on the other hand, exhibit delayed response times, background expression or leakiness, and challenges in achieving tight quantitative control. As a result, such transcriptional systems may be less suitable for applications requiring rapid, reversible control of gene function.^17,18^

**Table 1:** Non-Exhaustive List of Conditional Gene Expression and Post-Translational Conditional Regulation Systems Across Model Organisms

Level	Mechanism/System	Organisms	References	Component examples	Trigger/Inducers
DNA	Inducible promoters (transcriptional control)	Mammalian cells; Bacteria; Yeast; (also used in other organisms)	^45,46^	Tet-On/Tet-Off; PBAD promoter; Lac/IptG systems	Tetracycline, Doxycycline; arabinose; IPTG
DNA	Recombinase-based switches	Mammalian; *Drosophila*; Yeast; Bacteria; Mosquitoes	^47,48^	Cre/loxP (CreERT2); Dre/Rox	Tamoxifen (for CreERT2) or other ligands; temperature/chemical triggers
DNA	CRISPR-based transcriptional regulation (CRISPRa/CRISPRi)	Mammalian; Drosophila; *C. elegans*; Yeast; Bacteria	^49,50^	dCas9-VP64 (CRISPRa); dCas9-KRAB (CRISPRi)	Inducible promoters for dCas9; inducible gRNA expression; inducible dCas9 variants
DNA	Integrase-based gene toggles	Mammalian; *Drosophila*; Yeast; Bacteria; Mosquitoes	^51,52^	Site-specific integrases (e.g., Bxb1, phiC31)	Inducible triggers; signaling events
RNA	Riboswitches and riboregulators	Bacteria; Synthetic systems in yeast/mammals	^53,54^	Theophylline riboswitch; toehold switches	Theophylline; trigger RNAs
RNA	RNA interference and antisense strategies	Mammalian; *Drosophila*; *C. elegans*; Mosquitoes	^55,56^	inducible siRNA/shRNA; antisense RNAs	Inducible promoter expression of siRNA/shRNA or antisense
RNA	CRISPR-based RNA targeting/editing	Mammalian; *Drosophila*; *C. elegans*; Yeast; Bacteria	^19,57^	Cas13-based RNA targeting/editing	Inducible Cas13 expression or inducible gRNA delivery
RNA	RNA stability control elements	Bacteria; Synthetic contexts in yeast/mammals	^53,58^	RNA thermometers; AU-rich elements (AREs)	Temperature shifts or cellular conditions
Protein	Degron and degradation systems	Mammalian; Yeast; *Drosophila*; Mosquitoes	^4,27,59^	AID/OsTIR1; DD (destabilizing domains) with Shield-1	Auxin; Shield-1
Protein	Chemical-induced dimerization (CID)/reconstitution	Mammalian; *Drosophila*; Yeast; Bacteria	^4,44^	FRB/FKBP12; other CID pairs	Rapamycin or analogs
Protein	Optogenetic/light-controlled	Mammalian; *Drosophila*; *C. elegans*; Yeast; Bacteria	^34,60^	CRY2/CIB1; LOV domains	Light (specific wavelength)
Protein	Targeted degradation and activity control (PROTACs/molecular glues)	Mammalian	^61^	PROTACs; molecular glues	Small molecules

Post-transcriptional gene expression involves the regulation of RNA after its synthesis. Technologies such as RNA interference (RNAi) and RNA editing using CRISPR-based systems can modulate transcript abundance or sequence.^19^ In addition, engineered ribozymes and aptamers, which are ligand-responsive RNA elements, can be designed to promote mRNA cleavage or stabilization in the presence of specific small molecules. RNA aptamers can also be incorporated into engineered transcripts to alter RNA structure and regulate mRNA stability or translation in response to ligand binding^7,9,13,14^ (see Table 1 for RNA-level regulation systems). These systems can offer faster responses by acting on existing transcripts; however, their efficiency can vary depending on target accessibility and cellular context. They are important tools in functional genomics for gene knockdown and modulation of gene function.^20,21^ Despite these advantages, RNA-based regulatory approaches have their own limitations. RNAi-mediated effects may be transient due to the degradation of siRNAs, limiting the effectiveness of regulation.^22^ In addition, aptamers are susceptible to nuclease-mediated degradation and may trigger nonspecific immune responses, which can affect their stability and performance in vivo.^23^

In contrast, post-translational regulation enables direct control over protein stability and activity, providing rapid, reversible, and tunable modulation of biological function.^24^ These systems typically rely on engineered protein domains or degrons that respond to small molecules or physical stimuli, enabling conditional stabilization or degradation of target proteins.^24,25^ Because they act at the level of the functional molecule, post-translational systems are particularly well-suited for studying dynamic biological processes that require precise temporal resolution.^24,25^

To address these limitations, destabilizing domain (DD) systems have emerged as a powerful class of tools for post-translational control of protein function.^9,24^ In these systems, proteins of interest are fused to engineered domains that render them unstable and subject to proteasomal degradation in the absence of a ligand, but are stabilized upon ligand binding.^25^ The ligands can be grouped into two categories: small molecules and physical stimuli. Small molecule inducers include plant-derived compounds such as gibberellic acid^26^ and auxin,^27,28^ anticancer agents,^29^ antivirals,^30^ synthetic compounds such as Shield 1^4^, mifepristone (RU-486),^31^ and dTAG13^25^, and antibiotics such as trimethoprim.^32^ Physical stimuli inducers regulate protein function through cues such as light,^33–35^ temperature,^36,37^ oxygen,^38^ mechanical forces,^39,40^ and radiation.^41,42^ Some molecules, such as antibiotics and synthetic ligands, bind to fusion protein tags, thereby providing stability.^30^ In addition, there are degron systems, where target proteins are targeted for degradation through their respective ubiquitin pathways in response to ligands, such as the auxin-based degron system.^27^ (See Table 1 for protein regulation systems.)

Examples of such systems include the FKBP-DD system, which uses the FK506-binding protein FKBP12 destabilization domain fused to the protein of interest, and the ligand Shield-1, which binds to alter the abundance of the fused protein.^4,43^ This FKBP-DD system has been used in systems such as mammalian cells, mice, and parasites.^4,44^ The FKBP-DD is fused either to the N- or C-terminus of the protein of interest. When Shield-1 is introduced, FKBP-DD is stable, and the protein of interest does not degrade. This system is also reversible; however, the system is leaky,^6^ and the ligand Shield-1 is costly and toxic for long-term use.^8^ Each system, however, presents its own distinct limitations. Transcriptional and RNA-based methods may exhibit delayed kinetics or incomplete knockdown, whereas protein-level systems often require ligands or specialized conditions.

Another such system is the TMP-based *E. coli* dihydrofolate reductase (ecDHFR-DD) system. Among destabilizing domain systems, ecDHFR-DD is notable because of its advantages, such as reversibility and dose-dependent control, which must be balanced against considerations, including ligand properties such as solubility, cost, and potential off-target effects.

In this review, we focus on the TMP-based ecDHFR-DD system (hereinafter, referred to as DD), synthesizing current knowledge on its mechanism, performance across diverse biological systems, and key design considerations. We position this system within the broader landscape of conditional gene regulation technologies and discuss its applications across multiple fields, including functional genomics, synthetic biology, and biomedical research. We also consider its potential extension into applied biological contexts, such as pest and vector management, as part of a broader toolkit for conditional protein regulation.

### 1.1 Overview of EcDHFR-TMP Binding

Destabilizing domains are engineered protein domains that confer conditional stability to fused proteins in the presence of specific molecules or ligands.^4,24^ In the absence of the ligand, the fused protein is degraded, whereas ligand binding promotes its stabilization. Such systems are typically engineered through site-directed mutagenesis.^24^ The *E. coli* dihydrofolate reductase (DHFR) is a protein with an α/β structure, featuring eight-stranded β-sheets (strands A–H) and four flanking α-helices, mainly αB, αC, αE, and αF, that offer ligand-dependent stability and act as a DD when fused to the N- or C-terminal of the protein^62,63^ (Figure 1). The protein consists of the major subdomain and the adenosine-binding domain. The adenosine-binding subdomain is the smallest and serves as the binding site of the adenosine cofactor, while the major subdomain is made of residues from the N- and C-terminal with loops surrounding the active site.^63^

**Figure 1:**
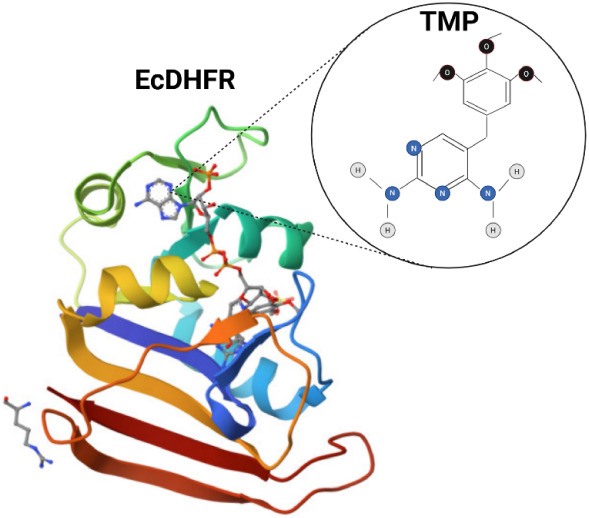
**Crystal structure of** *Escherichia coli* **dihydrofolate reductase (DHFR) in complex with trimethoprim (TMP)**. Ribbon representation of EcDHFR in complex with TMP, with a callout showing the chemical structure of TMP. Colored flat arrows represent the DHFR β-sheets, whereas α-helices are tightly coiled. Nicotinamide Adenine Dinucleotide Phosphate (NADPH) and trimethoprim are ligands that attach to pockets of the sheets and helices. The ribbon structure was extracted from the RCSB Protein Data Bank ID: 7MYM (https://www.rcsb.org/structure/7MYM), and the chemical structure of TMP was created using BioRender.com.

Trimethoprim (2,4-diamino-5-(3′,4′,5′-trimethoxybenzyl)-pyrimidine—TMP) is an inhibitor of DHFR (Figure 1). TMP is a clinically used antibiotic that inhibits bacterial growth by binding to bacterial DHFR, which catalyzes the NADPH-dependent reduction of dihydrofolate (DHF) to tetrahydrofolate (THF) and NADP^+^, a step involved in recycling folate.^64^ Inhibition of DHFR disrupts DNA replication due to its impact on folate-metabolizing enzymes.^65^ In the presence of TMP, the proteins fused to the domain are stabilized and escape degradation; however, this effect can be reversed as TMP is washed off (Figure 2). The use of this system involves designing N-terminal DD and/or C-terminal DD to stabilize the protein of interest and control its abundance. In some studies, N-terminal fusion fully stabilizes the protein at a narrow dose-response range, while the C-terminal fusion requires a higher ligand concentration for full stabilization.^24^ Some studies have suggested that the DD system works best when accessible to the proteasome; hence, cytoplasmic and nuclear proteins are effectively targeted,^66,67^ as compared to proteins in other compartments, although this appears to be controlled in other studies.^24^

**Figure 2:**
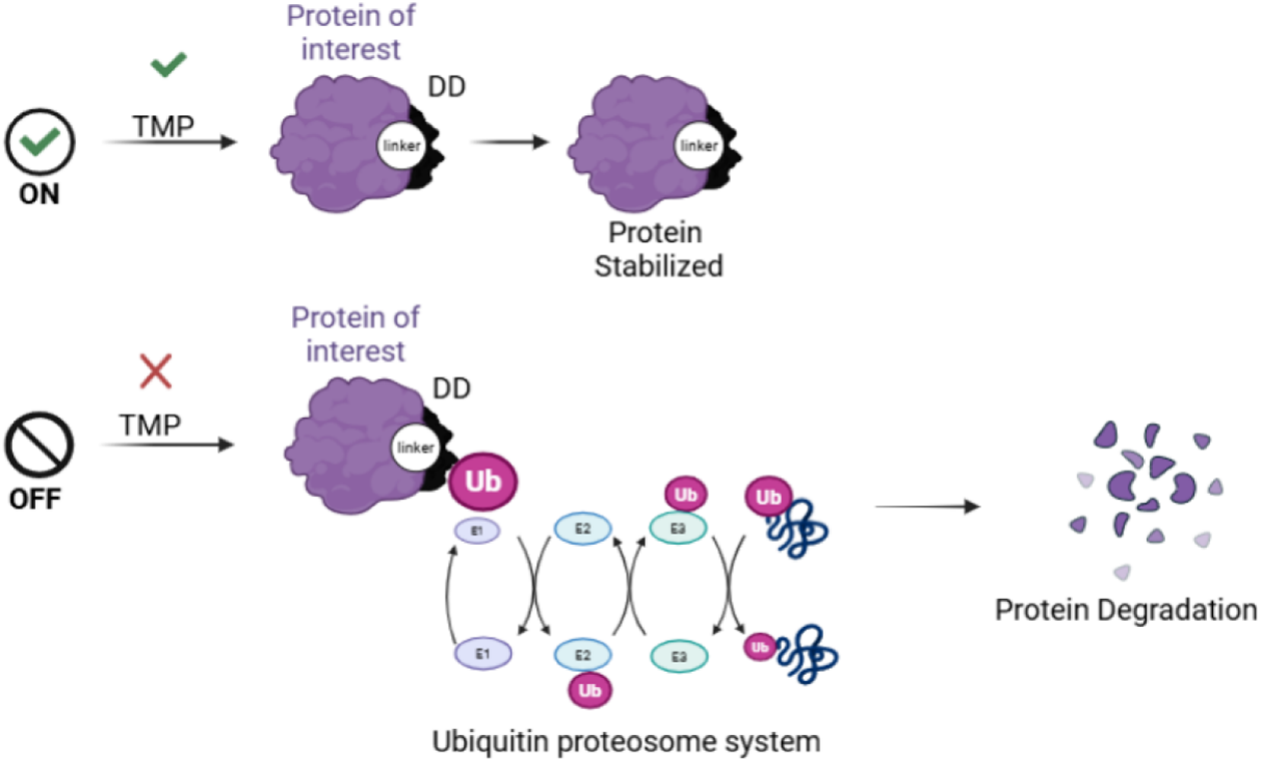
**TMP-based ecDHFR-DD system acting on the protein of interest.** The protein of interest is fused to an ecDHFR destabilizing domain (DD) via a linker. In the presence of trimethoprim (TMP) (ON state, top), TMP binds to the DD fusion protein and stabilizes its folded conformation, escaping the proteasome degradation pathways. In the absence of TMP (OFF state, bottom), the DD fusion remains conformationally unstable and is recognized by the ubiquitin–proteasome system, leading to ubiquitination of the fusion protein by E1, E2, and E3 enzymes, followed by proteasomal degradation. This system enables rapid and reversible ligand-dependent control of protein at the post-translational level.

Another consideration is the protein of fusion that is accessible to the proteasomal degradation pathway. The effectiveness of this DD system depends on stabilization by TMP or its analogs^4^ and degradation of the DD fusion protein mediated by the ubiquitin-proteasome system (UPS) in the absence of TMP, which involves the ubiquitin-activating enzyme E1, the ubiquitin-conjugating enzyme E2, and ubiquitin-protein ligase E3^68^. E1 catalyzes the binding of ubiquitin in an ATP-dependent reaction and then transfers it to E2, which subsequently brings ubiquitin to E3. This enables the transfer of ubiquitin to the targeted protein via an isopeptide bond, and repeated ubiquitination ultimately leads to protein degradation.^69^ The system has been reported most often in studies involving cytoplasmic and nuclear fusion proteins, where enzymes involved in the UPS are present.^65,66^ In contrast, other studies indicate poor regulatory stability in DD for proteins destined for the endoplasmic organelle or mitochondria,^70^ possibly resulting in lower accessibility to UPS pathways. However, this does not seem to be the case in other studies with similar targets.^71^ The host species and temperature also influence how the DD system works, since protein folding and degradation efficiency are affected by temperature.^59^ Most DDs are designed at 37 °C for warm-blooded animals; however, for organisms that prefer lower temperatures, temperature-optimized DDs that remain stable are required to enable clear TMP-dependent stabilization and low basal expression.^62^ Promoters, expression vectors, and the amount of DNA used are also crucial in understanding the expression system, and they may vary in activity across different cells, which should be considered in the ecDHFR-DD-POI regulation.^59^

## 2 ecDHFR Applications in Model Organisms

### 2.1 ecDHFR in Mammalian Systems

The TMP-based ecDHFR-DD system has been utilized in mammalian studies to investigate biological and chemical activities, demonstrating its applicability in conditional protein regulation. One such direction is the modulation of natural cellular pathways, with a focus on gene therapy, in which a drug can be used to control targeted genes and impact disease pathways.^68^ DDs have been used to regulate protein abundance in various parts of the mammalian system, including the brain.^71^ This regulatory system helps prevent detrimental effects from the overexpression of intended therapeutic genes. Of note, tetracycline systems have been used in brain-based gene therapy studies; however, these have been challenged by leakiness and immunogenicity, hence the need for improved systems to tightly control protein abundance.^13^ In this context, the DD system was used to examine biologically active proteins that are potential therapeutics, such as glial cell-derived neurotrophic factor (GDNF) in Parkinson’s disease, with rats as model systems.^71^ GDNF is essential for dopaminergic neurons lost in Parkinson’s, and this factor has potential for replenishing them. DD-based GDNF expression can be regulated by TMP in transgenic cells and in rat models in a dose-dependent, reversible manner.^71^ In inflammation studies, TMP was used to regulate the stability of the transcription factor nuclear factor κB (NFκB), which controls genes involved in inflammation and cell survival. NFκB is activated in response to microbial invasion, triggering inflammatory responses. However, unregulated NFκB activation is associated with pathological outcomes, including chronic inflammation and retinal degeneration.^66^ To fine-tune NFκB activity, researchers have fused the ecDHFR-DD to IκBα, an endogenous NFκB inhibitor. In the absence of TMP, the IκBα-DD protein is rapidly degraded, allowing NFκB signaling to proceed. Conversely, in the presence of TMP, the fusion protein is stabilized, inhibiting NFκB signaling and thereby attenuating inflammation.^66^ This system has also been used to understand the control of stress-response signaling,^67^ neuroprotection, and improvement of the system through testing different mutants, stabilizers, and TMP analogs.^59^

Gene editing technologies, such as CRISPR-Cas9, can also be controlled by the TMP-ecDHFR-DD system based on dose, time, and modulation of the involved genes.^72,73^ A study showed that fusing DD to transcription domains with engineered Cas9 complexes, followed by TMP in transient cells, enables transcription induction with upregulation of the mRNA and minimal transcription in the absence of TMP.^73^ Furthermore, transcription could be turned off after TMP removal,^73^ demonstrating that Cas9 activity can be temporarily tuned in this system. On the other hand, a technique called RESCUE RNA base editing is a potential strategy for treating genetic diseases and studying gene function; however, it is hindered by changes to RNA sequences.^74^ The ecDHFR-DD can be fused at the C-terminus of the RESCUE system and regulated by TMP. This combined system enabled control of RNA editing in a dose-dependent and reversible manner, as the protein was not degraded in the presence of TMP, and editing efficiency involving A-to-I and C-to-U was restored, reducing off-target effects.^74^

### 2.2 ecDHFR-DD in Apicomplexan Parasites, *Caenorhabditis elegans*, and Yeast Models

The DD system has been widely applied across various cell types and model organisms, including yeast (*Saccharomyces cerevisiae*) and *C. elegans*, to achieve conditional control of protein abundance. In these systems, temperature plays a critical role in modulating the stability of DD-tagged proteins, often influencing both the folding efficiency of DD and the activity of the proteasomal degradation machinery. Organisms such as zebrafish and *C. elegans* can thrive at relatively low temperatures, typically between 20–25 °C.^62^ Most DDs exhibit stable expression at 37 °C but decrease at lower temperatures; hence, optimization is required across different model organisms. To address this limitation, new DD mutants have been engineered to enable inducible regulation at lower temperatures in *C. elegans* studies.^62^ In these experiments, mutant libraries were generated by error-prone PCR and mutagenesis, and their expression was tested in the presence of trimethoprim (TMP) in a dose-dependent and reversible manner, demonstrating precise protein regulation in conditions closer to RT.^62^

The ecDHFR-DD system has also proven to be effective in apicomplexan parasites for functional analyses of genes involved in diverse biological processes. Because parasites such as *Plasmodium falciparum* are haploid during their developmental stages, DD systems offer a more efficient approach to gene function studies than knockout strategies.^75,76^ For instance, the DD system has been employed to investigate the role of Rpn6 in *P. falciparum*, which is a proteasome subunit involved in the degradation of ubiquitinated proteins. Rapid protein degradation was observed in the absence of TMP, while DD-tagged parasite lines remained viable in its presence.^76^ Similarly, DDs have been used to study the function of essential genes in *Cryptosporidium*.^77^ In this case, calcium-dependent protein kinase 1 (CDPK1), a potential drug target, was regulated by TMP, confirming its role in parasite proliferation.^77^

The ecDHFR-DD system has also been applied in yeast strains to control the target protein levels. While other domain systems, such as the FKBP-derived destabilizing domain, show instability in yeast, ecDHFR-DD displayed partial stabilization in the presence of TMP, although some background expression and leakiness were observed.^78^

### 2.3 ecDHFR-DD in *Drosophila melanogaster*

The ecDHFR-DD system has been shown to be active in manipulating protein stability in *Drosophila*.^7,32^ The DD variant used in these studies is derived from the mutant ecDHFR-DD engineered for low-temperature functionality and corresponds to a codon-optimized version of clone no. 7.^62^ This clone was selected on the basis of its high dynamic range, exhibiting low basal expression in the absence of trimethoprim (TMP) and strong stabilization upon ligand addition. These modifications enable the efficient performance of the DD system at temperatures compatible with *Drosophila* biology. For example, the DD system has provided valuable tools for exploring variations in proteasome activity among different cell types, although their effectiveness seems to vary across tissues.^32^ DDs have been successfully applied to manipulate neuronal circuits and to regulate the expression of membrane proteins such as ion channels and G-protein-coupled receptors.^32^ Of note, cellular TMP levels naturally decrease during pupation in *Drosophila*, making the system particularly effective in larval and adult stages, when feeding activity facilitates TMP uptake. TMP administration does not cause major adverse effects on fly survival or behavior; however, concentrations above 1 mM have been shown to delay development.^32^ The DD system can also be integrated with other existing *Drosophila* genetic tools, including the GAL4, GAL80, and FLP recombinase systems.^7,32^ For example, a GAL80-DD fusion can be activated by TMP in a dose-dependent and reversible manner to study neuronal activity in the brain, where varying TMP concentrations can be used to induce variable recombination frequencies in neuronal populations.^32^

The ecDHFR-DD system also enables the study of DNA double-strand break (DSB) repair in different chromatin contexts. In *Drosophila*, DSBs can be induced by expressing an I-SceI endonuclease fused to the DD domain; in the presence of TMP, the fusion protein remains stable, thereby preventing unwanted breaks during development.^79^ Additionally, the DD system has been applied to regulate gene-drive technologies such as CRISPR. In these approaches, gene-drive components, such as Cas9, are fused to DD, allowing their stabilization in the presence of TMP.^80^

## 3 Advantages and Limitations of the ecDHFR-DD System

TMP is commercially available and inexpensive, with good pharmacodynamic properties in diverse species. It is an antibiotic with a long history of use to treat and prevent infections in humans and is listed among essential drugs worldwide.^81^ It has a half-life of 8–10 h in humans.^65^ In most studies, it has been reported to have few off-target effects and can cross the blood–brain barrier and the placental barrier.^24^ This system works rapidly, in a dose-dependent manner, and is reversible by removing the TMP source, enabling genes to be turned on and off.^24^ Protein levels are reported to drop to very low levels when TMP is removed from the system. Despite the tightness of the system, studies have reported that some background protein levels, as part of the fused protein, may be produced when TMP is absent^62^ and may require system modifications, such as the use of dual DDs or modified ecDHFR mutants.^6^ Also, for the system to be effective, sufficient TMP concentrations should reach cells, and there should be an active proteasome in cells or tissues to enable efficient UPS degradation, especially in the absence of TMP.^32^ As with all conditional regulation systems, the utility of ecDHFR-DD depends on balancing its strengths with system-specific limitations. TMP is not very soluble in most solvents, but the most commonly used concentrations in assays are sufficient for protein stabilization in various organisms and cells; therefore, this should be taken into account when using the system across different organisms.^82^ TMP dissolves in DMSO; however, there are great impacts of the solvent on different organisms, and therefore, percentages of this solvent should be considered during feeding. In *Drosophila*, concentrations above 0.1% DMSO greatly affect larval fitness.^32^

TMP’s bacterial inhibition capacity disrupts gut microbiota differently according to the concentration used, and like any other molecule, overuse of TMP may result in antibiotic resistance, posing a threat to different organisms.^83^ Recent studies have shown substitute compounds for TMP that do not impact gut bacteria, such as 14a.^83^ However, the compound is less effective in some regions than TMP. Moreover, the compound has not been fully characterized and is not yet cleared as safe for use.^83^

While trimethoprim (TMP) has been widely used as a ligand for ecDHFR-based destabilizing domain (DD) systems, a broad range of structurally related compounds has been developed as dihydrofolate reductase (DHFR) inhibitors. These include TMP analogs and nonclassical antifolates such as pyrimethamine, cycloguanil, iclaprim, and tetroxoprim, many of which exhibit improved pharmacological properties, including altered binding affinity, selectivity, and solubility. However, the majority of these compounds have been characterized in the context of enzyme inhibition rather than post-translational protein stabilization.^65^ Recent studies have demonstrated that a subset of DHFR-binding molecules can function as pharmacological chaperones, stabilizing ecDHFR-DD fusion proteins, including several diaminopyrimidine- and triazine-based compounds validated in vitro and in vivo.^59^ Most of the identified molecules, such as ormetoprim, methotrexate, and diaveridine, are structurally similar to those of TMP and have been evaluated primarily in mammalian systems. Although these compounds can stabilize target proteins, they generally exhibit reduced potency or altered kinetic properties compared to TMP.^59^ Some molecules, such as methotrexate, exhibit additional toxicity that could impose cost constraints in large-scale applications.^84^ Importantly, solubility-enhanced derivatives, such as trimethoprim lactate and tetroxoprim, have not been systematically evaluated for their ability to stabilize ecDHFR-DD proteins. While the chemical space of DHFR-binding compounds is extensive, the subset of ligands validated for ecDHFR-DD remains underexplored.

## 4 Applied Contexts of Conditional Gene and Protein Regulation

Conditional gene expression and protein regulation systems are increasingly being explored across diverse biological contexts, including biomedical research, synthetic biology, biotechnology, and applied fields such as agriculture and public health.^17,18,24,32,85^ These systems provide powerful tools for manipulating gene function with spatial and temporal control, enabling both the development of applied strategies and the study of fundamental biological processes. Arthropods represent one important applied context due to their ecological, agricultural, and medical importance, demonstrating species that act as disease vectors in humans, livestock pests, and economically significant agricultural pests.^86^ Despite substantial advances in conditional gene regulation technologies in model organisms, their implementation in arthropods remains comparatively limited and represents an active area of research. Many existing systems have been adapted from model organisms such as *Drosophila* and mammalian systems, but their performance in arthropods can be constrained by factors including environmental variability, delivery methods, and regulatory precision.^32,86^

While these systems have broad relevance across biological disciplines, arthropods provide a particularly illustrative applied context in which conditional regulatory strategies are actively being explored. The increasing prevalence of insecticide resistance across arthropods of medical, agricultural, and veterinary importance has significantly reduced the effectiveness of conventional control strategies, posing challenges in both public health and agricultural systems.^87^ These arthropods include disease vectors, such as mosquitoes and ticks, livestock pests, such as flies and mites, and crop pests, such as lepidopteran and hemipteran species. In addition to resistance, reliance on chemical control negatively impacts nontarget organisms, threatens food security, and imposes economic burdens.^88^ These limitations have driven the development of alternative approaches, including genetic strategies that enable targeted and potentially sustainable control of insect populations.^89^

Current genetic control methods focus on population suppression or population modification.^90^ Population suppression aims to reduce insect abundance through methods such as the sterile insect technique (SIT) and other gene drive technologies.^90^ Population modification involves altering insect biological traits such as reproduction, behavior, or pathogen transmission, often replacing wild populations with modified ones, as seen with *Wolbachia*-based and transgene-based population modification.^91^

Central to these strategies is the ability to precisely regulate gene activity, thereby controlling proteins that influence survival and reproduction. Advances in genetic engineering have enabled the development of conditional gene expression and protein regulation systems that allow spatial, temporal, and inducible control of gene function.^92,93^ These tools offer opportunities to regulate traits detrimental to the engineered organism in a controlled, environmentally responsive manner. For example, conditional systems can be used to limit fitness, disrupt mating behaviors, or regulate the expression of essential proteins required for survival, thereby enhancing the precision of genetic control strategies.^85,94,95^ In particular, post-translational approaches, such as ligand-dependent protein stabilization systems, provide rapid and reversible control of protein activity, which may be advantageous in dynamic field environments.^93^

These technologies are increasingly being explored across diverse arthropod taxa, offering new opportunities for both fundamental biological research and applied pest management.^86^ As genetic engineering technologies continue to advance across insect taxa, integrating these regulatory systems into agricultural pest management and control strategies for insects of medical and veterinary importance represents a promising approach to reducing reliance on chemical insecticides while improving specificity and adaptability.

Among molecular tools, several conditional gene expression systems originally developed in model organisms have been adapted for use in arthropods. The GAL4-UAS system, originally developed in yeast, has been extensively applied in arthropods to study gene function and pathways. This system employs two independent transgenic lines: a driver line that expresses the yeast transcriptional activator GAL4 under a specific promoter, and a responder line that contains upstream activation sequences (UAS) that control the gene of interest.^96^ When the two lines are crossed, GAL4 binds to the UAS, initiating transcription of the target gene. In *Anopheles gambiae*, the GAL4-UAS system has been shown to enable gene function analysis by generating driver and responder lines via *piggyBac*-mediated transformation.^97^ Multitissue expression can also be achieved using this system, incorporating promoters that act ubiquitously, enabling phenotypic expression as well.^98^ GAL4 systems are advantageous in most insects, mainly because the driver and responder lines are easily assessed for feasibility and allow gene analysis based on phenotypes.^99^ In *Drosophila*, on the other hand, this system has been critical in expression studies of signaling pathways such as Hedgehog, highlighting its use in complex biological processes.^100^ The standard GAL4 system lacks intrinsic temporal regulation, which may be introduced by fusion with other regulators that are also leaky.^1^ To address these limitations, modified versions such as the *Gene Switch* system have been developed. This system uses a modified GAL4 system controlled by a progesterone analog, RU-486.^101^ In this version, the driver line expresses modified GAL4, and the responder line carries the UAS-target gene construct. The addition of RU-486 activates GAL4, enabling transgene expression, while its absence keeps the level of expression minimal. In *Aedes aegypti*, this system has been used to conditionally express GFP.^101^ However, even with these modifications, transcriptional systems can exhibit leaky expression, which may limit their precision in applications requiring tightly regulated gene activity.^102^

In transgenic studies, promoters also play a role in temporal control. *A. aegypti* heat shock protein (*hsp70*) activates transcription in different tissues, such as the midgut, in response to heat shock.^103^ This provides a basis for examining the expression associated with host–pathogen interactions as well. However, this system is challenged by the promoter’s natural leakiness. Furthermore, heat shock promoters lack spatial specificity; thus, there is a risk of ubiquitous expression. Because of the involvement of heat, some phenotypic changes may occur solely because of the heat treatment.^104^

The Q-system is another binary expression system that functions independently of GAL4 and Tet. It employs a QF driver line (expressing the QF transcriptional activator under a specific promoter) and a QUAS effector line, containing a gene of interest downstream of QUAS response elements.^14^ Here, the two lines are crossed, allowing QF to bind to QUAS and initiate transcription. Expression can be suppressed by the transcriptional repressor QS and restored upon addition of quinic acid (QA), which inhibits QS binding to QF.^1^ In *Anopheles gambiae* and *A. aegypti*, the Q-system has been used to understand neuronal circuits,^105^ cell labeling, and functional imaging using calcium sensors, which could help identify targets for vector control.^106^ This system, however, has faced the issue of QF toxicity, as observed in *Drosophila*,^14^ although a less toxic variant, QF2, has been developed and used in mosquito studies as well.^105^ Additionally, the CpG makeup in QUAS is often associated with gene silencing, hence affecting the expression ability of the system.^107^

The Tet system has also been used in arthropods of agricultural, medical, and veterinary importance^17,85,108,109^ for the expression of transgenes. This system consists of a driver line expressing a tetracycline-controlled transcriptional activator (*Tet)* and a responder line with a target gene under the control of tetracycline operator (*TetO*) sequences. Transcriptional control is achieved by modulating the interaction between the activator and *Tet*O in response to tetracycline or its analog, doxycycline.^45,110^ In the *Tet*-Off configuration, the activator binds *Tet*O and promotes transcription in the absence of tetracycline, while in the *Tet*-On version, the reverse activator binds TetO only in the presence of tetracycline.^110^ Because tetracycline readily crosses lipid membranes, it efficiently induces expression in most arthropod tissues. In *A. stephensi*, the *Tet* system has been established in lines by Minos-mediated germline transformation and using an *Anopheles gambiae* enhancer to control expression.^111^ Arthropod studies using the Tet system involve feeding tetracycline or doxycycline in sugar meals and larval diets and can be performed in a dose-dependent, reversible manner. A prominent application of this system is the release of insects carrying a dominant lethal (RIDL) in mosquitoes, as well as the self-limiting strains in agricultural pests.^85,112^ The *A. aegypti* strain OX513A and the self-limiting fall armyworm strain OX5382G are examples of strains that were developed and reared in the presence of tetracycline to suppress the lethal gene; upon release, males produce offspring that die in the absence of tetracycline.^85,108^ However, transcription-based systems are often limited by delayed response times, incomplete repression, and background expression, which can reduce their precision in applications requiring tight regulation.^17,85,108,109^

Within this context, the ecDHFR-DD system represents a promising tool for conditional protein regulation in arthropods and other nonmodel organisms. The DD could be fused to an essential protein in transgenic constructs, enabling conditional protein stabilization in the presence of trimethoprim (TMP) and targeted degradation upon its removal. Like other conditional systems, DD-tagged proteins can be integrated into transgenic strains, for example, in mosquitoes, to control effector genes for population suppression or modification. TMP supplementation during rearing ensures normal development, whereas withdrawal of TMP postrelease into the environment results in rapid degradation of these proteins, mainly during the next developmental cycle. This could induce lethality in field settings and enable controlled population suppression strategies, such as existing systems like RIDL or *Tet*-Off (Figure 3). Moreover, DDs could be fused with gene-drive components such as Cas9, or other nucleases or regulatory factors, so that drive activity becomes TMP-dependent, providing an additional post-translational layer in restricting gene-drive function to possible mass rearing and release settings.^80^

**Figure 3:**
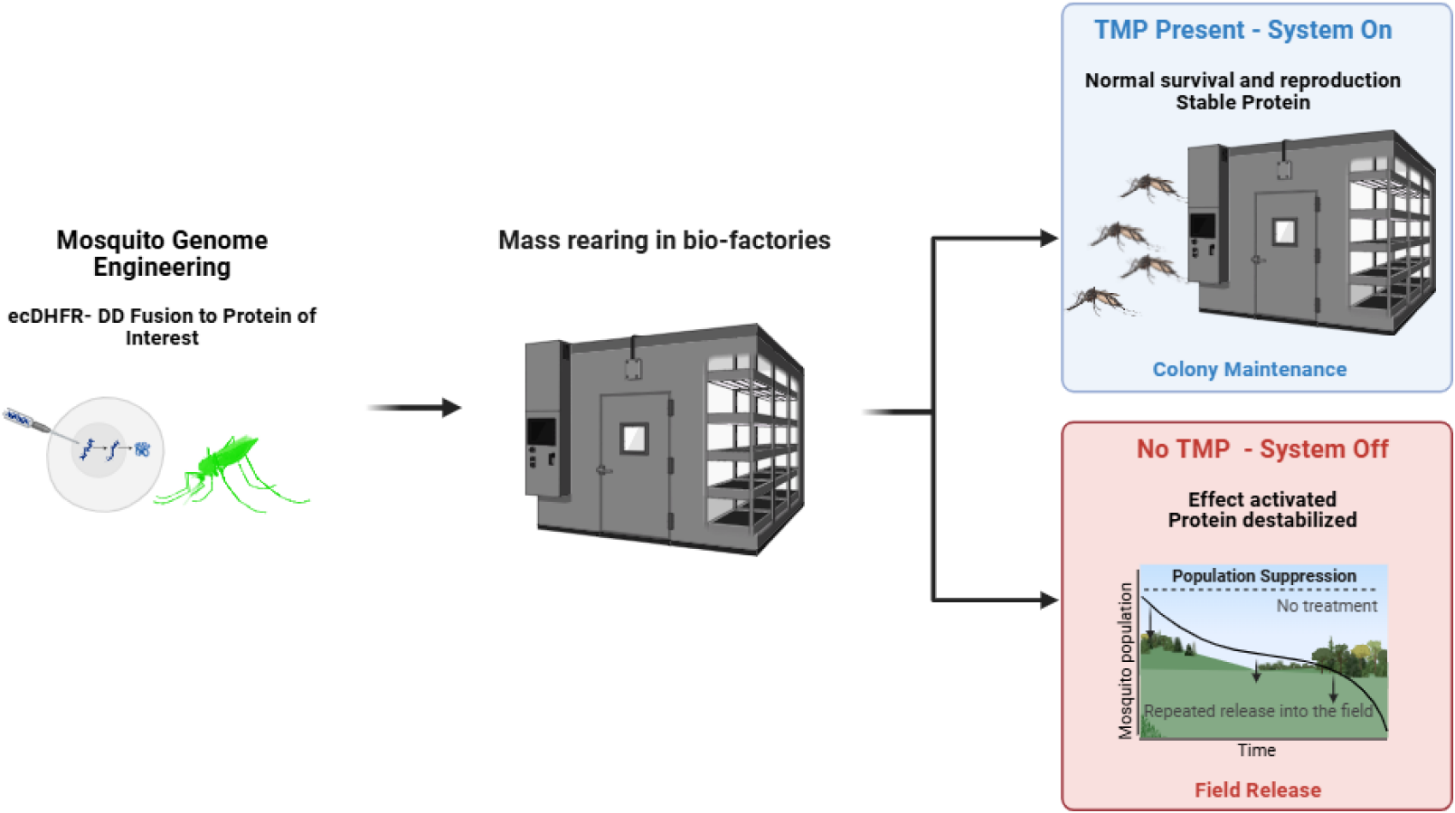
Example of a potential trimethoprim (TMP)-regulated ecDHFR destabilizing domain (DD) strategy for mosquito genome engineering and population control. A protein of interest is fused to ecDHFR-DD and genetically engineered into the mosquito genome. Transgenic mosquitoes are mass-reared in biofactories under TMP-supplemented conditions, where TMP stabilizes the DD-tagged protein, allowing normal survival, development, and reproduction for colony maintenance (“TMP present—system ON”). Upon release into the field, where TMP is absent, the DD-tagged protein becomes destabilized and degraded, activating the intended biological effect, such as reduced fitness or lethality, leading to progressive population suppression over time (“No TMP—system OFF”). Image created with Biorender.com.

Beyond population control, DDs could also be fused to immune factors, parasite interaction proteins, or salivary gland and midgut proteins that mediate key barriers to infection in vectors. This would enable temporal control of protein abundance at specific stages of infection, facilitating the functional analysis of transmission-blocking mechanisms. Similarly, fusion of DDs to developmental regulators or behavior-associated proteins could allow reversible, stage-specific control of gene function across larval, pupal, and adult stages, enabling studies of reproduction, physiology, and behavior without the lethality associated with permanent knockout. While these applications remain largely conceptual in many systems, they illustrate the broader potential of post-translational regulatory tools beyond traditional model organisms. Continued development and validation of these approaches across diverse taxa and biological systems will be essential to fully realizing their utility in both basic and applied biological research.

## 5 Conclusions

Current genetic control strategies for biological systems, including arthropods of medical, veterinary, and agricultural importance, increasingly leverage conditional gene expression systems to achieve the precise regulation of target genes. The ecDHFR destabilizing domain (DD) system is a powerful and versatile tool that enables post-translational control of protein stability and offers broad applications across diverse areas of research. Although this system has been successfully applied in *Drosophila* to modulate protein levels, its broader potential across diverse biological systems remains underexplored, representing a promising opportunity for further development across multiple disciplines.

A comprehensive understanding of how the ecDHFR-DD system functions across diverse organisms and biological contexts is essential to fully evaluate its capabilities and limitations. Key questions remain unanswered regarding its feasibility, dynamic range, reversibility, and fitness cost across both model and non-model systems. Understanding these areas will provide a foundation for broader application of the DD system in functional genomics, cell biology, and physiology, where temporal control of protein abundance is crucial.

Existing conditional expression systems, including widely used transcriptional regulatory approaches, often face critical limitations, such as leaky expression, incomplete target gene activation, and inconsistent performance across experimental and applied settings. These drawbacks compromise scalability and reliability, thereby undermining their effectiveness in research and translational contexts. The ecDHFR destabilizing domain (DD) system offers a promising alternative, providing tighter post-translational control of protein stability, minimizing off-target effects, and enhancing regulatory precision. Unlike transcriptional systems, the DD system directly links protein abundance to the presence of a small molecule ligand (TMP), enabling rapid, reversible, and dose-dependent modulation of gene function. To fully realize its potential, the ecDHFR-DD system should be systematically evaluated in cultured cell lines, model insects, and a broader range of biological systems, assessing its efficiency, stability, and compatibility with existing genetic tools (e.g., gene drives or CRISPR/Cas-based systems). Such studies will not only validate its utility for laboratory research but also pave the way for translational applications across synthetic biology, biomedical research, and applied biological systems, including vector and pest management. By expanding the application of the DD system within the broader genetic toolkit, researchers can achieve fine-tuned control over protein dynamics, addressing longstanding challenges in conditional gene regulation across diverse organisms and biological contexts.
